# 4-Bromo-2-(4-fluoro­benzyl­idene)indan-1-one

**DOI:** 10.1107/S1600536809025781

**Published:** 2009-07-18

**Authors:** Yi-Xin Zhou, Jian-Qiang Wang, Ren-Jun Du, Jian-Guo Tang, Cheng Guo

**Affiliations:** a College of Science, Nanjing University of Technology, Xinmofan Road No. 5 Nanjing, Nanjing 210009, People’s Republic of China

## Abstract

In the mol­ecule of the title compound, C_16_H_10_BrFO, the indane ring system is planar with a maximum deviation of 0.020 (3) Å. An intra­molecular C—H⋯O inter­action results in the formation of a planar ring, which is oriented at dihedral angles of 2.24 (3) and 2.34 (3)° with respect to the adjacent rings. π–π contacts between the benzene and indane rings [centroid–centroid distances = 3.699 (1) and 3.786 (1)Å] may stabilize the crystal structure.

## Related literature

For a related structure, see: Deeni & Ravi (2001 ▶). For bond-length data, see: Allen *et al.* (1987 ▶).
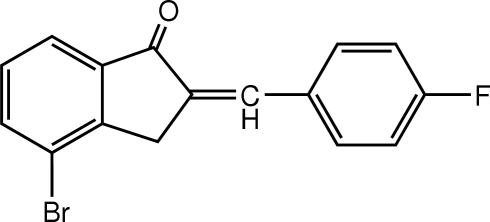


## Experimental

### 

#### Crystal data

C_16_H_10_BrFO
*M* *_r_* = 317.15
Triclinic, 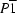

*a* = 7.3580 (15) Å
*b* = 7.4630 (15) Å
*c* = 13.140 (3) Å
α = 101.45 (3)°
β = 96.80 (3)°
γ = 111.72 (3)°
*V* = 642.2 (3) Å^3^
*Z* = 2
Mo *K*α radiation
μ = 3.20 mm^−1^
*T* = 294 K
0.10 × 0.10 × 0.05 mm

#### Data collection

Enraf–Nonius CAD-4 diffractometer
Absorption correction: ψ scan (North *et al.*, 1968 ▶) *T*_min_ = 0.740, *T*_max_ = 0.856
2518 measured reflections
2319 independent reflections
1351 reflections with *I* > 2σ(*I*)
*R*_int_ = 0.027
3 standard reflections frequency: 120 min intensity decay: 1%

#### Refinement

*R*[*F* ^2^ > 2σ(*F* ^2^)] = 0.059
*wR*(*F* ^2^) = 0.137
*S* = 1.00
2319 reflections
172 parameters
H-atom parameters constrained
Δρ_max_ = 0.38 e Å^−3^
Δρ_min_ = −0.35 e Å^−3^

### 

Data collection: *CAD-4 Software* (Enraf–Nonius, 1989 ▶); cell refinement: *CAD-4 Software*; data reduction: *XCAD4* (Harms & Wocadlo, 1995 ▶); program(s) used to solve structure: *SHELXS97* (Sheldrick, 2008 ▶); program(s) used to refine structure: *SHELXL97* (Sheldrick, 2008 ▶); molecular graphics: *ORTEP-3 for Windows* (Farrugia, 1997 ▶); software used to prepare material for publication: *SHELXL97* and *PLATON* (Spek, 2009 ▶).

## Supplementary Material

Crystal structure: contains datablocks global, I. DOI: 10.1107/S1600536809025781/hk2723sup1.cif

Structure factors: contains datablocks I. DOI: 10.1107/S1600536809025781/hk2723Isup2.hkl

Additional supplementary materials:  crystallographic information; 3D view; checkCIF report

## Figures and Tables

**Table 1:** Hydrogen-bond geometry (Å, °)

*D*—H⋯*A*	*D*—H	H⋯*A*	*D*⋯*A*	*D*—H⋯*A*
C3—H3*A*⋯O	0.93	2.14	2.972 (8)	149

**Fig. 1.**
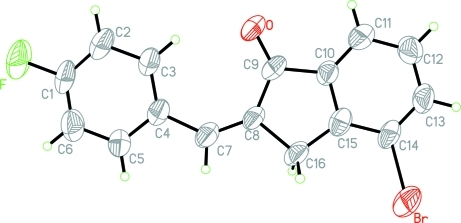
The molecular structure of the title molecule, with the atom-numbering scheme. Displacement ellipsoids are drawn at the 30% probability level.

## supplementary crystallographic information

### Comment

Some derivatives of 2,3-dihydro-1*H*-inden-1-one alcohol are important chemical materials. We report herein the crystal structure of the title compound.

In the molecule of the title compound (Fig 1), the bond lengths (Allen *et al.*, 1987) and angles are within normal ranges. Rings A (C1-C6), B (C8-C10/C15/C16) and C (C10-C15) are, of course, planar and the dihedral angles between them are A/B = 4.49 (3), A/C = 5.44 (3) and B/C = 0.96 (3) °. Intramolecular C-H···O interaction (Table 1) results in the formation of a planar ring (O/C3/C4/C7-C9/H3A), which is oriented with respect to the adjacent rings A and B at dihedral angles of 2.24 (3) and 2.34 (3) °, respectively. The indane ring system is planar with a maximum deviation of -0.020 (3) Å for atom C9. Atoms Br, O and C7 are 0.026 (3), -0.066 (3) and 0.075 (3) Å away from the plane of the indane ring system, respectively.

In the crystal structure, the π–π contacts between the benzene and the indane rings, Cg2—Cg1^i^ and Cg1—Cg3^ii^ [symmetry codes: (i) 1 - x, 2 - y, -z, (ii) 1 - x, 1 - y, -z, where Cg1, Cg2 and Cg3 are centroids of the rings A (C1-C6), B (C8-C10/C15/C16) and C (C10-C15), respectively] may further stabilize the structure, with centroid-centroid distances of 3.699 (1) and 3.786 (1) Å, respectively.

### Experimental

4-fluoro-benzaldehyde (10 mmol), 4-bromo-2,3-dihydro-1*H*-inden-1-one (10 mmol), anhydrous ethanol (10 ml) and 5 drops of piperidine were mixed in a three necked flask (50 ml). The flask was placed in a microwave synthesis system and irradiated for 7 min at 373 K with power 400 W. Then, the reaction mixture was slowly added with shaking to water (100 ml) and left to stand overnight. The precipitate was filtered, washed with water and dried (Deeni & Ravi, 2001). Crystals suitable for X-ray analysis were obtained by slow evaporation of an ethanol solution.

### Refinement

H atoms were positioned geometrically with C-H = 0.93 and 0.97 Å for aromatic and methylene H atoms, respectively, and constrained to ride on their parent atoms, with U_iso_(H) = 1.2U_eq_(C).

### Crystal data

**Table:**

C_16_H_10_BrFO	*Z* = 2
*M**_r_* = 317.15	*F*(000) = 316
Triclinic, *P*1	*D*_x_ = 1.640 Mg m^−^^3^
Hall symbol: -P 1	Mo *K*α radiation, λ = 0.71073 Å
*a* = 7.3580 (15) Å	Cell parameters from 25 reflections
*b* = 7.4630 (15) Å	θ = 9–13°
*c* = 13.140 (3) Å	µ = 3.20 mm^−^^1^
α = 101.45 (3)°	*T* = 294 K
β = 96.80 (3)°	Block, colorless
γ = 111.72 (3)°	0.10 × 0.10 × 0.05 mm
*V* = 642.2 (3) Å^3^	

### Data collection

**Table:**

Enraf–Nonius CAD-4 diffractometer	1351 reflections with *I* > 2σ(*I*)
Radiation source: fine-focus sealed tube	*R*_int_ = 0.027
graphite	θ_max_ = 25.3°, θ_min_ = 1.6°
ω/2θ scans	*h* = 0→8
Absorption correction: ψ scan (North *et al.*, 1968)	*k* = −8→8
*T*_min_ = 0.740, *T*_max_ = 0.856	*l* = −15→15
2518 measured reflections	3 standard reflections every 120 min
2319 independent reflections	intensity decay: 1%

### Refinement

**Table:**

Refinement on *F*^2^	Primary atom site location: structure-invariant direct methods
Least-squares matrix: full	Secondary atom site location: difference Fourier map
*R*[*F*^2^ > 2σ(*F*^2^)] = 0.059	Hydrogen site location: inferred from neighbouring sites
*wR*(*F*^2^) = 0.137	H-atom parameters constrained
*S* = 1.00	*w* = 1/[σ^2^(*F*_o_^2^) + (0.067*P*)^2^] where *P* = (*F*_o_^2^ + 2*F*_c_^2^)/3
2319 reflections	(Δ/σ)_max_ < 0.001
172 parameters	Δρ_max_ = 0.38 e Å^−^^3^
0 restraints	Δρ_min_ = −0.35 e Å^−^^3^

### Special details

**Table:**

Geometry. All e.s.d.'s (except the e.s.d. in the dihedral angle between two l.s. planes) are estimated using the full covariance matrix. The cell e.s.d.'s are taken into account individually in the estimation of e.s.d.'s in distances, angles and torsion angles; correlations between e.s.d.'s in cell parameters are only used when they are defined by crystal symmetry. An approximate (isotropic) treatment of cell e.s.d.'s is used for estimating e.s.d.'s involving l.s. planes.
Refinement. Refinement of *F*^2^ against ALL reflections. The weighted *R*-factor *wR* and goodness of fit *S* are based on *F*^2^, conventional *R*-factors *R* are based on *F*, with *F* set to zero for negative *F*^2^. The threshold expression of *F*^2^ > σ(*F*^2^) is used only for calculating *R*-factors(gt) *etc*. and is not relevant to the choice of reflections for refinement. *R*-factors based on *F*^2^ are statistically about twice as large as those based on *F*, and *R*- factors based on ALL data will be even larger.

### Fractional atomic coordinates and isotropic or equivalent isotropic displacement parameters (Å^2^)

**Table:**

	*x*	*y*	*z*	*U*_iso_*/*U*_eq_	
Br	0.47010 (11)	0.25337 (12)	0.59830 (5)	0.0759 (3)	
O	0.2276 (6)	0.2064 (7)	1.0423 (3)	0.0688 (12)	
F	0.8344 (6)	0.2939 (6)	1.4682 (2)	0.0883 (12)	
C1	0.7931 (11)	0.2919 (9)	1.3633 (4)	0.0618 (17)	
C2	0.6046 (11)	0.2666 (9)	1.3199 (4)	0.0657 (18)	
H2A	0.5071	0.2516	1.3604	0.079*	
C3	0.5620 (9)	0.2638 (9)	1.2136 (4)	0.0558 (16)	
H3A	0.4348	0.2475	1.1823	0.067*	
C4	0.7092 (9)	0.2854 (8)	1.1535 (4)	0.0483 (14)	
C5	0.8976 (9)	0.3109 (9)	1.2025 (4)	0.0586 (16)	
H5A	0.9979	0.3272	1.1639	0.070*	
C6	0.9387 (10)	0.3124 (9)	1.3084 (5)	0.0678 (18)	
H6A	1.0648	0.3273	1.3407	0.081*	
C7	0.6811 (9)	0.2884 (8)	1.0409 (4)	0.0543 (15)	
H7A	0.7986	0.3146	1.0156	0.065*	
C8	0.5240 (8)	0.2619 (8)	0.9659 (4)	0.0467 (14)	
C9	0.3131 (9)	0.2200 (8)	0.9680 (4)	0.0497 (15)	
C10	0.2123 (9)	0.1972 (8)	0.8595 (4)	0.0449 (13)	
C11	0.0132 (10)	0.1497 (9)	0.8219 (4)	0.0621 (17)	
H11A	−0.0777	0.1254	0.8663	0.075*	
C12	−0.0493 (10)	0.1387 (9)	0.7156 (4)	0.0642 (17)	
H12A	−0.1827	0.1093	0.6886	0.077*	
C13	0.0868 (10)	0.1714 (9)	0.6507 (4)	0.0607 (17)	
H13A	0.0442	0.1646	0.5800	0.073*	
C14	0.2830 (10)	0.2135 (8)	0.6883 (4)	0.0510 (15)	
C15	0.3494 (9)	0.2285 (8)	0.7946 (4)	0.0474 (14)	
C16	0.5512 (8)	0.2711 (9)	0.8538 (4)	0.0515 (15)	
H16A	0.6460	0.4025	0.8538	0.062*	
H16B	0.5987	0.1720	0.8228	0.062*	

### Atomic displacement parameters (Å^2^)

**Table:**

	*U*^11^	*U*^22^	*U*^33^	*U*^12^	*U*^13^	*U*^23^
Br	0.0960 (6)	0.1065 (6)	0.0373 (4)	0.0458 (5)	0.0289 (3)	0.0263 (3)
O	0.063 (3)	0.110 (4)	0.033 (2)	0.030 (3)	0.0189 (19)	0.022 (2)
F	0.117 (3)	0.104 (3)	0.0362 (19)	0.036 (3)	0.0016 (19)	0.0279 (19)
C1	0.085 (5)	0.061 (4)	0.029 (3)	0.022 (4)	0.000 (3)	0.011 (3)
C2	0.087 (5)	0.075 (5)	0.034 (3)	0.029 (4)	0.016 (3)	0.018 (3)
C3	0.063 (4)	0.070 (4)	0.033 (3)	0.028 (4)	0.005 (3)	0.011 (3)
C4	0.058 (4)	0.048 (4)	0.033 (3)	0.017 (3)	0.008 (3)	0.008 (3)
C5	0.055 (4)	0.068 (4)	0.041 (3)	0.017 (3)	0.003 (3)	0.009 (3)
C6	0.069 (5)	0.078 (5)	0.050 (4)	0.027 (4)	−0.005 (3)	0.019 (3)
C7	0.060 (4)	0.064 (4)	0.035 (3)	0.020 (3)	0.015 (3)	0.013 (3)
C8	0.052 (4)	0.058 (4)	0.028 (3)	0.020 (3)	0.013 (3)	0.010 (3)
C9	0.063 (4)	0.055 (4)	0.031 (3)	0.023 (3)	0.017 (3)	0.008 (3)
C10	0.053 (4)	0.052 (4)	0.032 (3)	0.024 (3)	0.009 (3)	0.010 (2)
C11	0.066 (5)	0.081 (5)	0.041 (3)	0.032 (4)	0.017 (3)	0.015 (3)
C12	0.063 (4)	0.087 (5)	0.047 (4)	0.040 (4)	0.004 (3)	0.010 (3)
C13	0.077 (5)	0.084 (5)	0.031 (3)	0.045 (4)	0.007 (3)	0.016 (3)
C14	0.077 (5)	0.055 (4)	0.028 (3)	0.033 (3)	0.015 (3)	0.011 (3)
C15	0.068 (4)	0.042 (3)	0.031 (3)	0.023 (3)	0.009 (3)	0.005 (2)
C16	0.060 (4)	0.060 (4)	0.030 (3)	0.018 (3)	0.016 (3)	0.011 (3)

### Geometric parameters (Å, °)

**Table:**

Br—C14	1.898 (6)	C8—C9	1.470 (8)
O—C9	1.224 (6)	C8—C16	1.521 (7)
F—C1	1.371 (6)	C9—C10	1.473 (7)
C1—C6	1.341 (9)	C10—C11	1.375 (8)
C1—C2	1.365 (8)	C10—C15	1.382 (7)
C2—C3	1.389 (7)	C11—C12	1.394 (7)
C2—H2A	0.9300	C11—H11A	0.9300
C3—C4	1.395 (8)	C12—C13	1.376 (8)
C3—H3A	0.9300	C12—H12A	0.9300
C4—C5	1.385 (7)	C13—C14	1.364 (8)
C4—C7	1.476 (7)	C13—H13A	0.9300
C5—C6	1.385 (7)	C14—C15	1.393 (7)
C5—H5A	0.9300	C15—C16	1.480 (7)
C6—H6A	0.9300	C16—H16A	0.9700
C7—C8	1.352 (7)	C16—H16B	0.9700
C7—H7A	0.9300		
			
C6—C1—C2	123.3 (5)	C8—C9—C10	107.1 (4)
C6—C1—F	118.5 (6)	C11—C10—C15	122.0 (5)
C2—C1—F	118.1 (6)	C11—C10—C9	128.3 (5)
C1—C2—C3	118.3 (6)	C15—C10—C9	109.7 (5)
C1—C2—H2A	120.9	C10—C11—C12	118.4 (6)
C3—C2—H2A	120.9	C10—C11—H11A	120.8
C2—C3—C4	120.3 (6)	C12—C11—H11A	120.8
C2—C3—H3A	119.8	C13—C12—C11	119.9 (6)
C4—C3—H3A	119.8	C13—C12—H12A	120.1
C5—C4—C3	118.4 (5)	C11—C12—H12A	120.1
C5—C4—C7	116.8 (5)	C14—C13—C12	121.2 (5)
C3—C4—C7	124.7 (5)	C14—C13—H13A	119.4
C4—C5—C6	120.9 (6)	C12—C13—H13A	119.4
C4—C5—H5A	119.5	C13—C14—C15	119.9 (5)
C6—C5—H5A	119.5	C13—C14—Br	121.3 (4)
C1—C6—C5	118.7 (6)	C15—C14—Br	118.8 (5)
C1—C6—H6A	120.7	C10—C15—C14	118.5 (5)
C5—C6—H6A	120.7	C10—C15—C16	111.1 (4)
C8—C7—C4	134.8 (5)	C14—C15—C16	130.3 (5)
C8—C7—H7A	112.6	C15—C16—C8	104.5 (4)
C4—C7—H7A	112.6	C15—C16—H16A	110.8
C7—C8—C9	132.6 (5)	C8—C16—H16A	110.8
C7—C8—C16	119.9 (5)	C15—C16—H16B	110.8
C9—C8—C16	107.5 (4)	C8—C16—H16B	110.8
O—C9—C8	129.6 (5)	H16A—C16—H16B	108.9
O—C9—C10	123.4 (5)		
			
C6—C1—C2—C3	−0.6 (10)	O—C9—C10—C15	177.6 (5)
F—C1—C2—C3	−179.8 (5)	C8—C9—C10—C15	−1.8 (6)
C1—C2—C3—C4	0.3 (9)	C15—C10—C11—C12	−1.8 (9)
C2—C3—C4—C5	−0.5 (9)	C9—C10—C11—C12	179.1 (5)
C2—C3—C4—C7	−178.8 (5)	C10—C11—C12—C13	1.2 (9)
C3—C4—C5—C6	0.8 (9)	C11—C12—C13—C14	0.4 (10)
C7—C4—C5—C6	179.3 (5)	C12—C13—C14—C15	−1.4 (9)
C2—C1—C6—C5	1.0 (10)	C12—C13—C14—Br	178.7 (5)
F—C1—C6—C5	−179.9 (5)	C11—C10—C15—C14	0.8 (9)
C4—C5—C6—C1	−1.1 (10)	C9—C10—C15—C14	180.0 (5)
C5—C4—C7—C8	176.9 (6)	C11—C10—C15—C16	−178.2 (5)
C3—C4—C7—C8	−4.8 (11)	C9—C10—C15—C16	0.9 (7)
C4—C7—C8—C9	0.7 (12)	C13—C14—C15—C10	0.9 (8)
C4—C7—C8—C16	−178.4 (6)	Br—C14—C15—C10	−179.3 (4)
C7—C8—C9—O	3.4 (11)	C13—C14—C15—C16	179.7 (6)
C16—C8—C9—O	−177.5 (6)	Br—C14—C15—C16	−0.5 (8)
C7—C8—C9—C10	−177.3 (6)	C10—C15—C16—C8	0.2 (6)
C16—C8—C9—C10	1.9 (6)	C14—C15—C16—C8	−178.7 (5)
O—C9—C10—C11	−3.3 (10)	C7—C8—C16—C15	178.0 (5)
C8—C9—C10—C11	177.4 (6)	C9—C8—C16—C15	−1.3 (6)

### Hydrogen-bond geometry (Å, °)

**Table:**

*D*—H···*A*	*D*—H	H···*A*	*D*···*A*	*D*—H···*A*
C3—H3A···O	0.93	2.14	2.972 (8)	149
